# Chronic Morel-Lavallée Lesion in a Pediatric Patient: An Underrecognized Sequela after Trauma

**DOI:** 10.1155/2023/6662079

**Published:** 2023-11-21

**Authors:** Anthony O. Kamson, Bradley Lazzari, Joshua Murphy

**Affiliations:** ^1^UPMC Central PA, Harrisburg, PA, USA; ^2^Children's Healthcare of Atlanta, Atlanta, GA, USA

## Abstract

Morel-Lavallée lesions are serious internal degloving injuries associated with trauma. Its diagnosis and treatment can be challenging. We describe the surgical treatment of a case of a chronic Morel-Lavallée lesion in a pediatric patient who sustained an injury to her left thigh during an all-terrain vehicle accident more than a year ago.

## 1. Introduction

Morel-Lavallée lesions in the pediatric population remain relatively understudied and underappreciated [1]. Morel-Lavallée lesions (MLL) are serious internal degloving injuries associated with trauma. Shearing forces between subcutaneous fat and underlying fascia result in injury to blood vessels and lymphatics. Hemolymphatic fluid can then accumulate in the zone of injury. If left untreated, the underlying fluid collection can lead to pain, disability, skin necrosis, and sepsis. Most of these lesions occur in the adult population, with a limited number of pediatric cases being reported [1–3]. To our knowledge, there is a paucity of literature on the surgical management of MLL in pediatric patients. We describe a case of surgical treatment of a chronic MLL in a pediatric patient following failure of extensive nonsurgical management. The patient and legal guardian provided written informed consent for the publication of this case report.

## 2. Case Presentation

**Figure 1:**
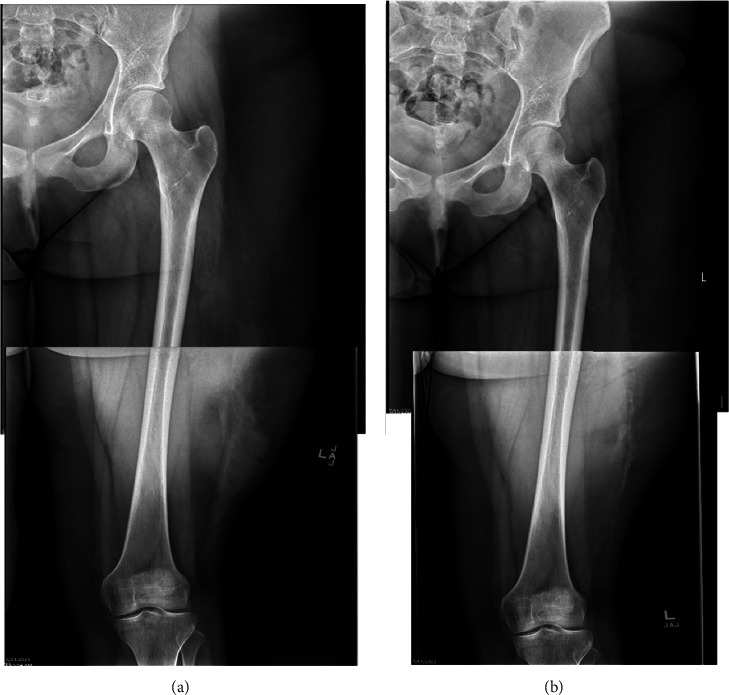
Initial AP femur radiograph of a 17-year-old female who sustained an ATV accident. Significant soft tissue swelling is noted laterally without any fracture. (a) Initial injury and (b) 13 months post-injury.

**Figure 2:**
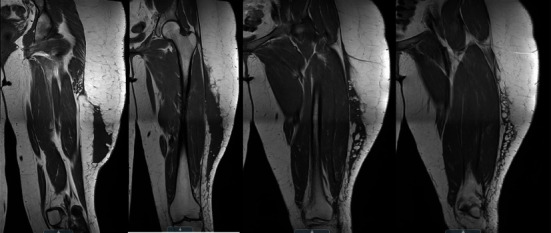
MRI T1 sequence of the left thigh approximately 1 month post-injury demonstrating large fluid collection with fat globules superficial to the deep fascia of the left thigh measuring 24.2 cm × 3.9 cm.

**Figure 3:**
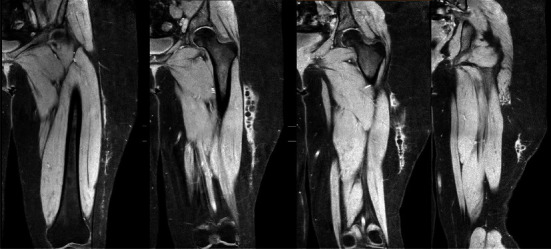
MRI T2 sequence of the left thigh approximately 13 months post-injury demonstrating decreased size of the lateral fluid collection superficial to the deep fascia of the left thigh.

A 17-year-old female with no significant past medical history presented to the outpatient office for left thigh pain and swelling. Approximately 13 months prior to this presentation, she had sustained a blunt injury to left side of her body after an all-terrain-vehicle accident where she collided with a tree. Following the initial accident, she had significant pain and swelling of the left thigh. She was seen at an emergency department the day after the accident, where X-rays were negative (Figure 1) and no further advanced imaging was obtained. One month later, due to continued thigh pain, an MRI was obtained which demonstrated a Type V Morel-Lavallée lesion of the lateral thigh with a pseudonodular appearance, internal enhancement, and a thin capsule (Figure 2). Initial treatment included conservative measures, with ice, compression, and physical therapy. Over the course of one year, the patient experienced revolving symptom improvement and worsening, despite extensive conservative treatment including compressive dressings, NSAID's, and physical therapy. A repeat MRI was obtained 13 months after the initial incident and demonstrated the lesion had decreased slightly in size but had formed a pseudocapsule (Figure 3). After an extensive discussion, a shared decision was made to proceed with surgical management of the Morel-Lavallée lesion.

**Figure 4:**
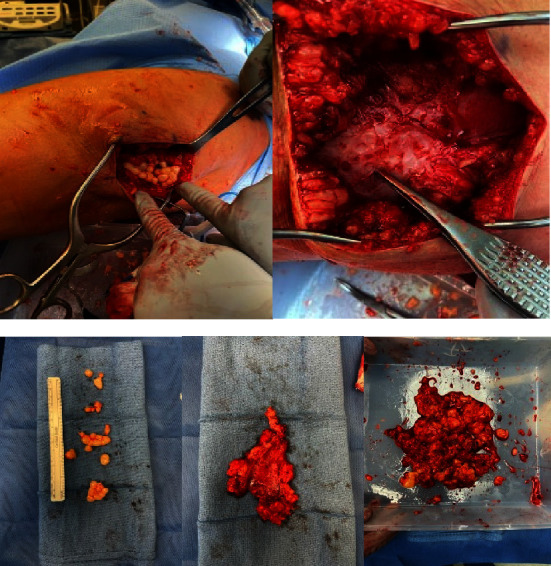
Clinical photographs of lateral fluid collection superficial to the deep fascia of the left thigh. Fat globules were encountered and a hardened pseudocapsule encapsulated with fatty tissue was excised.

A longitudinal direct lateral incision approximately 15 cm was made directly over the lesion, which was easily palpable. Soft tissue dissection was carried down until a cystic cavity was encountered, which appeared to be adhered to the iliotibial band and subcutaneous tissue. The cavity was incised, and multiple fat globules were encountered and excised along with the pseudocapsule in its entirety (Figure 4). Tissue cultures were sent to the laboratory for further analysis. The cavity was irrigated with a pulse lavage and 3 liters of normal saline. Two Jackson-Pratt drains were placed in the dead space and the wound was closed in a layered fashion. An elastic wrap was placed with slight compression. The patient had an uneventful postoperative course and was discharged from the hospital on postoperative day one. Operative cultures were negative for any microorganism colonization. The Jackson-Pratt drains and compressive ACE wrap remained in place for 2 weeks and were removed without complication. Her left thigh pain and swelling finally resolved and she remained symptom-free as noted at her 1 year postoperative follow-up.

## 3. Discussion

MLL are serious soft tissue internal degloving lesions secondary to shearing forces that are associated with traumatic injures [4]. These injuries were first described in the 1800's by the French surgeon Maurice Morel-Lavallée. The understanding of the pathophysiology and the ability to promptly recognize and diagnose these lesions has improved significantly over time and has led to improved treatment strategies and patient outcomes [5]. Historically, MLL have often been misdiagnosed or underappreciated due to the subtle presentation and lack of specific symptoms [6]. With the development of advanced imaging techniques such as ultrasound, computed tomography, and magnetic resonance imaging, prompt identification and treatment of these lesions have become more feasible [7].

Initially, the majority of these lesions were treated with an aspiration or irrigation and debridement, which unfortunately led to high recurrence rates and multiple complications [8]. However, due to early recognition, treatment has evolved and minimally invasive techniques have been developed. Conservative management, which is usually utilized for smaller lesions, includes compressive dressings, serial aspirations, and negative pressure wound therapy. When conservative measures fail or if a large lesion is present, an open irrigation and debridement may be required to prevent chronic pain, cellulitis, or abscess formation which could lead to severe complications such as an amputation [9, 10]. Currently, there is no established gold standard as all treatment modalities have variable success rates and treatment is determined by the severity and characteristics of the lesion [11].

However, imaging findings can be subtle or nonspecific in children, therefore requiring a high index of suspicion to prevent delayed diagnosis [12]. In pediatric patients, MLL MLL most commonly occurs following an acute traumatic event which can range from sports injuries, falls, or motor vehicle accidents. Compared to the adult population, these lesions are more commonly secondary to sport injuries and are more commonly located in the hip, thigh, knee, and leg [13, 14]. Diagnosis of MLL begins with a thorough history and physical exam. A differential diagnosis for MLL includes but is not limited to myositis, hematoma, or fat necrosis [5, 15]. The mechanism of injury is particularly important to raise suspicion for a shearing force. On physical exam, patients may present with soft tissue swelling, skin hypermobility, decreased cutaneous sensation, or fluctuance. In a third of patients, symptoms may present in a delayed fashion [14]. Diagnosing MLL in pediatric patients can be difficult due to the rarity of the condition and similarities to other soft tissue abnormalities. MRI has become the gold standard to confirm the diagnosis as it best demonstrates the separation of the subcutaneous fat from the fascial plane [14]. Mellado and Bencardino proposed an MRI classification system of MLL that includes 6 types of lesions based on the shape, signal enhancement, and the presence of a capsule [7].

Early recognition and appropriate management are crucial in preventing complications associated with MLL. Initial conservative treatment is usually reserved for acute small lesions (<50 cm^3^) without underlying fractures. Treatment consists of compression bandage, NSAIDs, and physiotherapy. High patient compliance is required for successful nonoperative management [16]. Serial clinical assessments and imaging should be used to monitor improvement. When conservative measures fail to alleviate symptoms, percutaneous aspiration or open surgical intervention may be necessary to prevent the formation of a chronic cavity or infection [5, 16]. Sclerotherapy is an adjuvant to percutaneous drainage with talc, alcohol, or doxycycline injected into the dead space followed by percutaneous drainage. Sclerotherapy can be used as a first-line therapy in patients with acute lesions that are refractory to compressive dressings and in patients with chronic lesions. In chronic lesions, percutaneous drainage may result in recurrent postoperative hematoma or secondary infection; therefore, it is mandatory to combine sclerotherapy with percutaneous drainage with sclerotherapy [17–20]. Otherwise, an open irrigation and debridement is the gold standard for surgical intervention when either a large lesion is present (>50 cm^3^), conservative treatment has failed to alleviate symptoms, or when a pseudocapsule has formed [21].

MLL in pediatric patients pose a unique challenge compared to adults as children have different healing capacities and their growth plates may be affected by the lesion or by surgical management [22]. It is important to carefully monitor growth plate integrity and to consider long term complications when determining the appropriate treatment. The most common complication seen in treating MLL is recurrence. These lesions have approximately a 56% reoccurrence rate with nonoperative management and a 15% reoccurrence rate in operative management. Chronic, untreated lesions can result in pseudocyst formation, which increases the risk of infection. Skin necrosis is another potential complication from delayed treatment or serial debridement. In the pediatric population, there is an increased frequency of fatal complications due to children being vulnerable to higher shock per unit area and having incomplete development of subcutaneous fat leading to more severe degloving injuries [23, 24].

## 4. Summary

Morel-Lavallée lesions are a rare but significant injury in the pediatric population. These lesions are difficult to diagnose and are commonly missed. Determining if nonsurgical or surgical treatment is appropriate is complex. Physicians should include MLL in their differential diagnosis of patients with trauma, and occasionally even in the absence of a shearing injury.

## Data Availability

The data used to support the findings of this study are available on request as it includes HIPAA confidential information. Please contact the corresponding author for further information.

## References

[1] Kushare I., Ghanta R. B., Wunderlich N. A. (2020). Morel-Lavallée lesions (internal degloving injuries) of the lower extremity in the pediatric and adolescent population. *The Physician and Sportsmedicine*.

[2] Remien K. A., Khemka S., Person A., Mike T. B. (2023). Delayed onset of pediatric morel-lavallée lesion: a case report. *Journal of Orthopaedic Case Reports*.

[3] Zhou Y., Wang B., Wang J., Zhang M. (2023). A case report of severe unstable pelvic fracture with extensive perineal destruction and morel-lavallee lesion, with literature review. *International Journal of Surgery Case Reports*.

[4] Mooney M., Marshall G., Drew K., Hanna M., Ebraheim N. (2020). Surgical treatment of a chronic morel-lavallée lesion: a case report. *Journal of Orthopaedic Case Reports*.

[5] Singh R., Rymer B., Youssef B., Lim J. (2018). The morel-lavallée lesion and its management: a review of the literature. *Journal of Orthopaedics*.

[6] Diviti S., Gupta N., Hooda K., Sharma K., Lo L. (2017). Morel-lavallee lesions-review of pathophysiology, clinical findings, imaging findings and management. *Journal of Clinical and Diagnostic Research: Journal of Clinical and Diagnostic Research*.

[7] Mellado J. M., Bencardino J. T. (2005). Morel-Lavallée lesion: review with emphasis on MR imaging. *Magnetic Resonance Imaging Clinics of North America*.

[8] Shen C., Peng J.-P., Chen X.-D. (2013). Efficacy of treatment in peri-pelvic morel–lavallee lesion: a systematic review of the literature. *Archives of Orthopaedic and Trauma Surgery*.

[9] Carlson D. W. A., Simmons J., Sando W., Weber T., Clements B. (2007). Morel-lavalée lesions treated with debridement and meticulous dead space closure: surgical technique. *Journal of Orthopaedic Trauma*.

[10] Shimizu T., Matsuda S., Sakuragi A., Tsukie T., Kawanabe K. (2015). Simultaneous occurrence of a severe morel-lavallée lesion and gluteal muscle necrosis as a sequela of transcatheter angiographic embolization following pelvic fracture: a case report. *Journal of Medical Case Reports*.

[11] Hussein K., White B., Sampson M., Gupta S. (2019). Pictorial review of morel-lavallée lesions. *Journal of Medical Imaging and Radiation Oncology*.

[12] Boushnak M. O., Rabah H., Saleh M. H., Aaraj G. A., Hajjar S., Moussa M. K. (2021). Post-traumatic late presentation of morel–lavallée: case report and review of literature. *Journal of Orthopaedic Case Reports*.

[13] Goller S. S., Erber B., Ehrnthaller C., Ricke J., Armbruster M. (2022). Peripelvic morel-lavallée lesion following high-energy spine trauma: case report and review of treatment options. *Trauma case reports*.

[14] Mazingi D., Jakanani G. C., Mushayavanhu P. (2018). Morel-Lavallée lesion in a 12-month-old child: a case report and literature review. *International Journal of Surgery Case Reports*.

[15] Divjak N., Kwiatkowski B., Tercier S. (2019). Morel-Lavallée lesion of the knee in the young athlete. *Pediatric Emergency Care*.

[16] Molina B. J., Ghazoul E. N., Janis J. E. (2021). Practical review of the comprehensive management of morel-lavallée lesions. *Plastic and Reconstructive Surgery-Global Open*.

[17] Bansal A., Bhatia N., Singh A., Singh A. K. (2013). Doxycycline sclerodesis as a treatment option for persistent morel-lavallée lesions. *Injury*.

[18] Luria S., Yaakov A., Yoram W., Meir L., Peyser A. (2006). Talc sclerodhesis of persistent morel-lavall??E lesions (posttraumatic pseudocysts). *Journal of Orthopaedic Trauma*.

[19] Penaud A., Quignon R., Danin A., Bahé L., Zakine G. (2011). Alcohol sclerodhesis: an innovative treatment for chronic morel-lavallée lesions. *Journal of Plastic, Reconstructive and Aesthetic Surgery*.

[20] Tran W., Foran J., Wang M., Schwartz A. (2008). Postsurgical bleeding following treatment of a chronic Morel-Lavallée lesion. *Orthopedics*.

[21] Gupta A., Kumar V., Agarwal A., Suresh A. (2021). Management of recurrent post-traumatic seroma of thigh (Morel-Lavallée lesion) by percutaneous aspiration and sclerotherapy using tetracyclines (past). *BMJ Case Reports*.

[22] Shelley J., Noritake A., Ortiz K., Ricca R. (2017). Morel-lavallee lesion in pediatric trauma. *Pediatric Surgery International*.

[23] Rha E. Y., Kim D. H., Kwon H., Jung S.-N. (2013). Morel-lavallee lesion in children. *World Journal of Emergency Surgery*.

[24] Lee K. J. (2008). Initial stabilization in severely injured child. *Journal of the Korean Medical Association*.

